# Predictive factors of functional movement screening and physical fitness in university students: the roles of gender, activity level, and sleep duration

**DOI:** 10.3389/fpubh.2026.1776073

**Published:** 2026-04-13

**Authors:** Samir Abdelnaby Shaaban Essa, Mona Metwally El-Sayed, Ayman Mohamed El-Ashry, Mahmoud Abdelwahab Khedr, Amira Abdelhamid Shawky Morsy, Sameer Mohammed Sayyd, Wadee Abdullah Alkuhaili, Osama Ibrahim Abukhaizaran, Raghad Kamal Tarwneh, Boshra Karem Mohamed El-Sayed, Shaimaa Mohamed Amin, Lamiaa Abd El Hakeem Ali Ahmed, El-Saied Abd El-Hamid El-Sayed Salem

**Affiliations:** 1 Department of Sports Sciences and Physical Activity, Faculty of Education, Taibah University, Madinah, Saudi Arabia; 2 Department of Community, Psychiatric, and Mental Health Nursing, College of Nursing, Qassim University, Buraydah, Saudi Arabia; 3 Psychiatric and Mental Health Nursing, Faculty of Nursing, Alexandria University, Alexandria, Egypt; 4 College of Sciences and Humanities, Health and Physical Education Department, Prince Sultan University, Riyadh, Saudi Arabia; 5 Nursing Administration Department, Faculty of Nursing, Alexandria University, Alexandria, Egypt; 6 Nursing Department, North Private College of Nursing, Arar, Saudi Arabia; 7 Faculty of Nursing, Psychiatric Mental Health Nursing Department, Cairo University, Cairo, Egypt; 8 Department of Fitness, Gymnastics, and Sports Shows, Faculty of Sports Sciences for Men, Alexandria University, Alexandria, Egypt

**Keywords:** health promotion, motor activity, physical fitness, sex factors, sleep, students, wounds and injuries

## Abstract

**Background:**

Functional movement and physical fitness are critical indicators of health and performance in young adults, yet their determinants among university students remain underexplored.

**Objective:**

This study examined the predictive roles of gender, age, physical activity level, sleep duration, injury history, and academic performance on functional movement screening (FMS) scores and physical fitness outcomes.

**Methods:**

A cross-sectional design was employed among university students. Functional movement was assessed using the FMS, while physical fitness was measured through standardized strength, endurance, and flexibility tests. Multiple regression analyses were conducted to identify significant predictors.

**Results:**

Gender emerged as the strongest predictor across both domains: females achieved higher FMS scores, whereas males demonstrated superior physical fitness, particularly in strength and endurance. Physical activity level significantly predicted functional movement but not overall physical fitness, highlighting qualitative versus quantitative dimensions of performance. Age was positively associated with muscular strength, while low GPA was unexpectedly linked to higher physical fitness. Recent injury history (<6 months) negatively influenced movement efficiency, whereas sleep duration and smoking status were not significant predictors when controlling for other variables.

**Conclusion:**

Findings underscore the multidimensional nature of student health, with gender, activity level, and injury history serving as primary determinants. These results highlight the need for integrated, gender-sensitive, and injury-prevention strategies to optimize movement quality and physical fitness in university populations.

## Introduction

Physical fitness is a multidimensional construct that significantly influences overall health and well-being, encompassing cardiovascular endurance, muscular strength, flexibility, and body composition (1). Maintaining physical fitness is particularly important for university students, who often face sedentary lifestyles, academic pressures, and lifestyle changes that can negatively impact physical performance and health outcomes, including obesity, stress, and reduced physical activity (2, 3).

The Functional Movement Screen (FMS) is a widely accepted tool for assessing fundamental movement patterns, mobility, stability, balance, and neuromuscular control (4, 5). It identifies movement deficiencies and guides interventions aimed at improving physical fitness and reducing injury risk. While the FMS has been extensively studied in athletic populations, less is known about its application in non-athletic university students, highlighting a gap this study seeks to address (6, 7).

Previous research indicates that gender, physical activity level, and sleep duration influence functional movement and physical fitness. Males generally exhibit higher muscular strength and cardiovascular fitness, while females demonstrate greater flexibility (8, 9). Regular physical activity is associated with improved cardiovascular health, muscular strength, and functional movement, whereas inadequate activity and poor sleep duration may impair physical performance and recovery (10–12).

The biopsychosocial model provides a framework to examine the interplay between biological, psychological, and social factors affecting physical fitness, making it suitable for investigating predictors of functional movement in university students (13). Integrating gender, activity level, and sleep duration allows for a multidimensional understanding of functional movement and physical fitness, which can inform targeted interventions tailored to the unique needs of this population.

### Significance of the study

This study extends existing knowledge by simultaneously examining multiple predictors of functional movement and physical fitness in a non-athletic university population. By exploring the relationships between gender, activity level, and sleep duration, the study provides a holistic perspective on factors influencing physical performance. Findings can guide the development of health promotion programs, injury-prevention strategies, and movement-based interventions that address the specific needs of university students. Ultimately, this research contributes to improved physical fitness, reduced injury risk, and enhanced well-being and academic performance among young adults.

Although variables such as gender, physical activity, sleep, and injury history have been widely studied, they are often examined independently rather than within an integrated framework. Few studies have simultaneously modeled Functional Movement Screening (FMS) and multidimensional physical fitness outcomes using advanced statistical approaches (e.g., generalized linear models) in non-athletic university populations. Addressing this gap, the present study adopts a multidimensional approach to examine the combined effects of these factors on functional movement and physical fitness among university students.

### Aim of the study

This study aimed to investigate the predictive roles of gender, physical activity level, and sleep duration on functional movement screening scores and physical fitness outcomes among university students.

### Research hypotheses

H_1_: Gender will significantly predict functional movement and physical fitness outcomes among university students.

H_2_: Higher levels of physical activity will be positively associated with improved functional movement and physical fitness performance compared to sedentary or moderately active students.

H_3_: Sleep duration will significantly predict functional movement performance, with students reporting adequate sleep (≥8 hours per night) exhibiting higher functional movement scores than those with insufficient sleep.

H_4_: Previous injury history will negatively influence functional movement performance, with students who experienced a recent injury (<6 months) compared to those without an injury history.

## Methods

### Study design and setting

This descriptive, cross-sectional study followed the Strengthening the Reporting of Observational Studies in Epidemiology (STROBE) guidelines to ensure methodological rigor. The study was conducted at the Department of Sports Sciences and Physical Activity, Faculty of Education, Taibah University, Madinah, Saudi Arabia. This department offers comprehensive academic programs in physical education, exercise science, and health promotion, and provides a structured environment for both teaching and applied research in human performance. It is equipped with state-of-the-art laboratories and specialized facilities, including motion analysis systems, physiological testing equipment, and standardized tools for functional movement screening. These resources enabled the accurate and reliable assessment of physical fitness and functional movement outcomes, thus supporting the validity of the study findings.

### Sample size and sampling procedure

Sample size estimation was performed using G Power version 3.1.4 (14), with linear multiple regression specified as the statistical test. Based on an assumed medium effect size (*f*^2^ = 0.15), a significance level of 0.05, and a desired statistical power of 0.95, the minimum required sample was calculated to be 194 participants. To accommodate possible non-response or attrition, the target sample size was increased to 220 students. Of these, 11 students declined participation, yielding a final sample of 209 participants and a response rate of 95%. Participants were recruited through a convenience sampling approach, focusing on students who fulfilled the inclusion criteria and were accessible within the university environment.

The study population consisted of undergraduate students enrolled at Taibah University, Madinah, Saudi Arabia. The inclusion criteria were restricted to students who provided written informed consent and were free from acute or chronic musculoskeletal disorders that could influence physical performance or compromise the validity of functional movement assessments. Exclusion criteria included a history of recent surgery, diagnosed neurological disorders, or any physical limitations that could impede the safe completion of physical fitness tests. In addition, professional athletes and students engaged in specialized or high-intensity training programs were excluded to ensure sample homogeneity and reduce the risk of performance bias.

### Study instruments

#### The demographic form

A structured demographic form was used to collect background information from participants. The form included variables such as age, gender, and GPA. It also collected data on lifestyle-related factors, including physical activity level, previous injury history, sleep duration, and smoking status.

#### Physical activity level

It was assessed using the International Physical Activity Questionnaire (IPAQ) short form, a widely used and standardized self-report instrument developed for cross-national surveillance of health-related physical activity in adults. IPAQ measures activity across multiple domains (walking, moderate-intensity, and vigorous-intensity activity) over the last 7 days and allows categorization into low, moderate, and high activity levels using established scoring protocols based on frequency, duration, and intensity (i.e., MET-minutes/week criteria). Moderate activity is defined by a combination of days and minutes of walking or moderate-intensity activity (e.g., ≥30 min on ≥5 days) or a total activity of ≥600 MET-minutes/week, while high activity requires vigorous activity on ≥3 days totalling ≥1,500 MET-minutes/week or any combination of walking, moderate, and vigorous activity achieving ≥3,000 MET-minutes/week. Participants who did not meet criteria for moderate or high activity were classified as sedentary/low active. The IPAQ has been tested for reliability and validity in multiple international settings, demonstrating acceptable measurement properties for population-level physical activity assessment, making it suitable for our study sample of university students (15, 16).

### Functional movement screen

The FMS is a standardized assessment tool developed by Gray Cook et al. (17) to evaluate fundamental movement patterns essential for physical performance. It aims to identify functional limitations, asymmetries, or weaknesses that could increase the risk of injury or compromise movement efficiency. The FMS provides a reliable method for screening mobility, stability, balance, and neuromuscular control in a structured and systematic way. The FMS consists of seven specific test components, each designed to assess a distinct aspect of movement quality. These include:

*Deep squat*: this movement assesses total body mechanics, with a particular focus on ankle and hip mobility, as well as thoracic spine and shoulder stability. It is a comprehensive test of flexibility and coordination in a weight-bearing posture.

*Hurdle step*: this test examines single-leg balance and the ability to maintain alignment and control while stepping over a mid-height obstacle. It challenges the coordination between the hips, knees, and ankles while testing core stability.

*In-line lunge*: this movement assesses lower limb stability and balance while challenging the alignment and control of the torso and lower extremities. It simulates the dynamic deceleration and control required during sports and daily activities.

*Active straight-leg raise*: this test measures hamstring flexibility and the ability to dissociate movement of one leg while maintaining pelvic control and core stability. It helps identify restrictions in posterior chain mobility.

*Trunk stability push-up*: this component evaluates core strength and trunk stability during a pushing motion. It requires the participant to maintain a rigid, aligned position of the spine and pelvis throughout the test.

*Rotary stability*: this complex test assesses neuromuscular coordination and stability during diagonal and rotational movements. It challenges the participant’s ability to maintain control while performing contralateral reach from a quadruped position.

*Shoulder mobility test*: this test measures the range of motion and coordination of the upper extremities, with a particular focus on shoulder flexibility, thoracic spine mobility, and symmetrical movement.

Each of the seven test components is scored on a 4-point scale: a score of 3 indicates that the movement was completed correctly without compensation, a score of 2 indicates that the movement was completed with compensation, a score of 1 indicates that the movement could not be performed, and a score of 0 indicates that the participant experienced pain during the movement. The total possible score is 21, and a score of 14 or lower has been associated with an increased risk of injury.

The FMS has demonstrated acceptable levels of validity and reliability in evaluating functional movement patterns. Cook et al. (17) developed the FMS to identify movement deficiencies and predict injury risk. Regarding reliability, Kiesel et al. (42) reported excellent interrater reliability, with an intra-class correlation coefficient (ICC) of 0.98, and strong interrater reliability, with an ICC of 0.87, among professional raters. The tool has also shown good predictive validity for injury risk, particularly in athletic populations. For example, a total FMS score of ≤14 has been associated with a significantly higher likelihood of injury, with sensitivity values ranging from 0.54 to 0.72 and specificity values between 0.60 and 0.82, depending on the population studied (18, 42).

In this study, the FMS was administered by trained assessors according to standardized protocols to ensure safety and scoring reliability. The assessment was used to evaluate the functional movement patterns of university students and to investigate their association with gender, physical activity level, sleep duration, and overall physical fitness. In the current study, the internal consistency of the FMS was assessed using Cronbach’s alpha, which yielded a reliability coefficient of 0.881, indicating a high level of internal consistency among the test components in this sample of university students.

### Physical fitness assessments

The physical fitness assessment in this study incorporated a battery of standardized field-based tests that evaluated key components of health-related fitness, as shown in Table 1. Flexibility was measured using the sit-and-reach test, a widely accepted assessment of hamstring and lower back flexibility (19). Cardiovascular fitness was indirectly assessed through resting heart rate, recorded after 5 minutes of seated rest, which is a reliable indicator of baseline cardiovascular efficiency and conditioning (20). Muscular endurance was evaluated using the push-up test, which requires participants to perform continuous repetitions until fatigue, providing a valid and reliable measure of upper-body endurance (21). Finally, muscular strength was assessed using a handgrip dynamometer, with grip strength recognized as a simple yet powerful predictor of overall muscular strength and long-term health outcomes (22). Together, these assessments provided a comprehensive, practical, and validated toolset for evaluating multiple dimensions of fitness, flexibility, cardiovascular status, muscular endurance, and muscular strength, among university students.

**Table 1:** Descriptive statistics for physical fitness variables in the research sample (*n* = 209).

Variables	Min	Max	Mean	±SD	Skewness	Kurtosis
Sit and reach test	23.00	55.00	32.00	7.23	0.78	0.14
Resting heart rate test	60.00	140.00	91.03	17.27	0.47	−0.28
Push-up test	0.00	58.00	25.85	11.98	0.30	−0.47
Handgrip strength test (right hand)	7.00	38.00	23.21	8.30	0.17	−1.21
Handgrip strength test (left hand)	5.00	38.00	22.26	8.09	0.16	−1.26

### Study procedures

#### Tools preparation and pilot study

To ensure the suitability and reliability of the instruments used in this study, careful tool preparation was undertaken. The FMS was selected as the primary assessment tool for evaluating participants’ fundamental movement patterns. The FMS is a widely validated and standardized tool developed by Cook et al. (17), with established reliability in diverse populations. It consists of seven specific movement tasks designed to assess mobility, stability, and neuromuscular control. The selection of the FMS was appropriate, given its strong alignment with the study’s objectives of evaluating physical fitness and functional movement, as well as its relevance to the university student population. Trained assessors administered the tool according to standardized procedures to ensure consistency and accuracy in scoring.

Prior to launching the full study, a pilot study was conducted with 20 students from the target population (excluded from the final analysis) to assess the clarity of instructions, feasibility of the assessments, and time required. Feedback confirmed that the procedures were practical and no modifications were necessary. The psychometric properties of the FMS were then evaluated. Internal consistency was examined using the pilot sample data, yielding a Cronbach’s alpha of 0.881, indicating high reliability among the seven test components.

Although the Functional Movement Screen (FMS) consists of distinct movement tasks rather than items forming a unidimensional psychometric scale, Cronbach’s alpha was calculated as a descriptive indicator of internal consistency. It is acknowledged that the FMS is primarily a performance-based assessment tool, and Cronbach’s alpha should be interpreted cautiously in this context.

Subsequently, the structural validity of the FMS was tested in the full sample (*N* = 209) using Confirmatory Factor Analysis (CFA). A single-factor model, with all seven movement components loading onto a latent “Functional Movement” factor, demonstrated acceptable fit: *χ*^2^(14) = 28.64, *p* = 0.012; CFI = 0.94; TLI = 0.91; RMSEA = 0.07 (90% CI: 0.03–0.10); SRMR = 0.05. All factor loadings were statistically significant (*p* < 0.001) and ranged from 0.52 to 0.79. These findings support the unidimensionality of the FMS and justify the use of a total composite score in this university student sample. Prior to regression analysis, multicollinearity was assessed. All Variance Inflation Factor (VIF) values were within acceptable limits (range: 1.02 to 2.34), confirming that predictor variables were not unduly correlated. Residual plots were examined and supported the appropriateness of the Generalized Linear Models.

### Data collection

Data collection was conducted over two months, following the completion of tool preparation and the pilot study. Participants were recruited from the Department of Sports Sciences and Physical Activity at Taibah University. After obtaining informed consent, students who met the inclusion criteria were scheduled for assessments in designated university facilities under standardized conditions. Each participant completed the demographic questionnaire, followed by the FMS and physical fitness assessments. Trained assessors conducted all evaluations to ensure consistency, accuracy, and participant safety. The data collection process was organized to accommodate student schedules and minimize disruptions to academic activities. All collected data were recorded immediately and securely stored to maintain confidentiality and data integrity throughout the study period.

### Resting heart rate measurement protocol

Resting heart rate (RHR) was measured as an indicator of cardiovascular fitness. Participants were instructed to avoid caffeine, vigorous exercise, and heavy meals for at least 2 h prior to assessment. Measurements were taken in a seated position after a 5-min rest period in a quiet environment. Heart rate was recorded using a validated digital heart rate monitor, and three consecutive readings were taken and averaged to determine each participant’s final resting heart rate. Variability in RHR may also reflect individual differences, stress levels, or lifestyle factors common in university populations. These clarifications provide context for interpreting the RHR data and support its use as an indicator of cardiovascular fitness. Measurements were conducted at consistent times of day to minimize circadian variation.

### Data analysis

The collected data were reviewed, coded, and analyzed using IBM SPSS v25 and AMOS v24. Descriptive statistics, including means, standard deviations, and frequency distributions, were used to summarize the data, while percentages illustrated categorical variables. Prior to hypothesis testing, the structural validity of the FMS was evaluated using Confirmatory Factor Analysis (CFA). A single-factor model was specified, with the seven FMS components loading onto a latent “Functional Movement” factor. Model fit was assessed using multiple indices: the chi-square statistic (*χ*^2^), the comparative fit index (CFI), the Tucker-Lewis index (TLI), the root mean square error of approximation (RMSEA) with its 90% confidence interval, and the standardized root mean square residual (SRMR). Acceptable model fit was defined as CFI and TLI ≥ 0.90, RMSEA ≤ 0.08, and SRMR ≤ 0.08. The reliability of the FMS was further confirmed using Cronbach’s alpha. To examine group differences, independent samples t-tests compared outcomes between genders, while one-way ANOVA assessed variations across activity levels and sleep categories, followed by post-hoc tests (e.g., Tukey’s HSD) where significant differences were found. Given the multiple comparisons conducted, we acknowledge the potential for Type I error inflation; therefore, unexpected or isolated findings are interpreted conservatively and considered exploratory rather than confirmatory.

Generalized Linear Models (GLMs) were used to examine the predictive relationships between independent variables (gender, age, physical activity level, sleep duration, injury history, and GPA) and outcomes (FMS scores and physical fitness T-scores). For continuous outcomes, a Gaussian distribution with an identity link function was specified.

Model assumptions were assessed prior to analysis: independence was ensured by study design, multi collinearity was evaluated using Variance Inflation Factors (VIFs), with all values below 5 (range: 1.02–2.34), and residual diagnostics supported model adequacy. Model fit was evaluated using deviance statistics, and given evidence of over dispersion (Deviance/df > 1), robust standard errors were applied. Results are reported as regression coefficients with 95% Wald confidence intervals, along with Wald Chi-square statistics. Standardized beta (*β*) coefficients were calculated to provide effect size estimates. All tests were two-tailed, with statistical significance set at *p* ≤ 0.05.

## Results

Table 2 shows that the majority of students were above 18 years (69.9%), with a mean age of 18.28 ± 0.43 years. Females constituted 56.9% of the sample. Academically, most students achieved high performance, with 63.2% obtaining a GPA of 4.0–5.0, while only 0.5% scored below 2. Regarding lifestyle factors, 71.3% engaged in moderate physical activity, 27.3% in high activity, and 1.4% were sedentary. Nearly half (48.8%) reported no previous injuries, while 17.2% had injuries dating back more than 12 months. Sleep duration was predominantly within 4–8 h per night (75.1%), with 23.9% exceeding 8 h. Smoking prevalence was low, with only 7.7% identified as smokers.

**Table 2:** Demographic and lifestyle characteristics of the participants.

Personal and academic data	*N* = 209	%
Age		
≤18 years	63	30.1
>18 years	146	69.9
Min–Max	17.50–19.20	
Mean ± SD	18.28 ± 0.43	
Gender		
Male	90	43.1
Female	119	56.9
GPA		
D (<2)	1	0.5
C (2–<3)	12	5.7
B (3–<4)	64	30.6
A (4–5)	132	63.2
Physical activity level		
Sedentary	3	1.4
Moderately active	149	71.3
Highly active	57	27.3
Previous injury history		
No injury	102	48.8
<3 Months	37	17.7
3–<6 Months	22	10.5
6–<9 Months	5	2.4
9–<12 Months	7	3.3
≥12 Months	36	17.2
Sleeping hours		
Less than 4 h	2	1.0
4–8 h	157	75.1
More than 8 h	50	23.9
Smoking		
Yes	16	7.7
No	193	92.3

Table 3 shows that participants demonstrated generally high functional movement capacity, with a mean total FMS score of 16.06 ± 4.02 (76.46% of the maximum). Most students were classified in the high (70.33%) or moderate (29.19%) performance categories, while only 0.48% scored low. Among the individual components, the highest scores were observed in Rotational Stability, Trunk Stability, and Hurdle Step (>80%), whereas Deep Squat (67.94%) and Shoulder Stability (64.91%) recorded the lowest scores, with 24.88 and 30.62% of participants, respectively, falling in the low category. These findings highlight overall good functionality, with specific limitations in mobility and upper-body stability.

**Table 3:** Frequency and percentage for functional movement FMS variables under consideration by levels (N = 209).

Variables	Levels						Mean ± SD	Mean score percent
	Low		Moderate		High			
	*N*	%	*N*	%	*N*	%		
Deep squat	52	24.88%	93	44.50%	64	30.62%	2.04 ± 0.78	67.94%
Hurdle step	36	17.22%	50	23.92%	123	58.85%	2.42 ± 0.77	80.54%
In-line lunge	28	13.40%	78	37.32%	103	49.28%	2.36 ± 0.71	78.63%
Straight leg	33	15.79%	76	36.36%	100	47.85%	2.32 ± 0.73	77.35%
Trunk stability test on wooden planks	44	21.05%	28	13.40%	137	65.55%	2.44 ± 0.82	81.50%
Rotational stability	23	11.00%	52	24.88%	134	64.11%	2.53 ± 0.69	84.37%
Shoulder stability test	64	30.62%	91	43.54%	54	25.84%	1.95 ± 0.76	64.91%
Total functional movement (FMS)	1	0.48%	61	29.19%	147	70.33%	16.06 ± 4.02	76.46%

Table 1 indicates that participants demonstrated moderate-to-good physical fitness overall. Flexibility averaged 32.00 ± 7.23 cm, with most values clustering in the lower-to-moderate range. Resting heart rate was relatively high at 91.03 ± 17.27 bpm, with some participants exceeding normal levels. Muscular endurance showed wide variability, with a mean of 25.85 ± 11.98 push-ups, while handgrip strength was balanced between hands (right: 23.21 ± 8.30 kg; left: 22.26 ± 8.09 kg), reflecting average bilateral performance without significant dominance.

Table 4 reveals significant associations between participants’ characteristics and physical fitness outcomes. Age was an important factor, as students older than 18 years demonstrated superior muscular endurance and strength, reflected in higher push-up scores and greater handgrip strength (*p* < 0.01). Gender differences were particularly marked, with males outperforming females in flexibility, muscular endurance, handgrip strength, and cardiovascular efficiency. Notably, males recorded significantly lower resting heart rates (*p* < 0.001), indicating better baseline cardiovascular conditioning. Academic performance also correlated with physical fitness, where students with higher GPAs showed lower resting heart rates but weaker push-up and grip strength performance, while those with lower GPAs displayed greater muscular strength and endurance (*p* < 0.001). Lifestyle factors further influenced fitness outcomes. Highly active participants achieved better cardiovascular profiles and upper-body performance compared to their less active peers (*p* < 0.05), while sedentary individuals unexpectedly recorded the highest push-up scores, likely due to outliers. Injury history negatively affected performance, with recently injured students showing reduced strength and endurance (*p* < 0.05). Sleep duration did not significantly impact fitness measures. However, smoking status revealed unexpected patterns: smokers had lower resting heart rates yet demonstrated higher push-up and grip strength performance (*p* < 0.01), suggesting potential confounding influences or subgroup characteristics.

**Table 4:** Relationship between physical fitness variables and personal and academic data.

Personal and academic data	Sit and reach test	Resting heart rate test	Push-up test	Handgrip strength test (right hand)	Handgrip strength test (left hand)
	Mean ± SD	Mean ± SD	Mean ± SD	Mean ± SD	Mean ± SD
Age					
≤18 years	31.02 ± 6.53	94.13 ± 17.41	21.97 ± 10.72	20.24 ± 6.98	19.46 ± 6.92
>18 years	32.42 ± 7.49	89.70 ± 17.10	27.53 ± 12.13	24.49 ± 8.52	23.47 ± 8.27
Test of sig. (p)	T = 1.295(0.197)	T = 1.709(0.089)	T = 3.144(0.002) *	T = 3.483(0.001) *	T = 3.373(0.001)
Gender					
Male	35.07 ± 7.41	80.36 ± 11.07	34.98 ± 10.35	31.56 ± 3.92	30.53 ± 3.54
Female	29.68 ± 6.18	99.11 ± 16.75	18.95 ± 7.78	16.89 ± 4.07	16.01 ± 3.77
Test of sig. (p)	T = 5.725(0.000) *	T = 9.208(0.000) *	T = 12.784(0.000) *	T = 26.190(0.000) *	T = 28.335(0.000) *
GPA					
D (<2)	37.00 ±	75.00 ±	22.00 ±	25.00 ±	25.00 ±
C (2–<3)	36.00 ± 6.66	80.83 ± 13.93(a)	33.75 ± 11.66(b)	30.58 ± 4.32(b)	29.50 ± 4.70(b)
B (3–<4)	31.97 ± 7.03	88.25 ± 16.13(ab)	30.19 ± 12.89(ab)	25.44 ± 8.66(a)	24.52 ± 8.35(a)
A (4–5)	31.61 ± 7.33	93.43 ± 17.64(b)	23.06 ± 10.66(a)	21.44 ± 7.83(a)	20.49 ± 7.61(a)
Test of sig. (p)	*F* = 2.039(0.133)	*F* = 4.328(0.014) *	*F* = 11.379(0.000) *	*F* = 10.945(0.000) *	*F* = 11.463(0.000) *
Physical activity level					
Sedentary	31.33 ± 7.51	97.67 ± 23.25(a)	35.33 ± 10.97(a)	28.00 ± 6.08(a)	26.67 ± 5.86(a)
Moderately active	31.88 ± 7.23	92.93 ± 17.05(a)	24.44 ± 11.79(a)	22.01 ± 8.26(a)	21.15 ± 7.98(a)
Highly active	32.35 ± 7.34	85.72 ± 16.72(a)	29.05 ± 11.85(a)	26.09 ± 7.82(a)	24.93 ± 7.86(a)
Test of sig. (p)	*F* = 0.100(0.905)	*F* = 3.930(0.021) *	*F* = 4.137(0.017) *	*F* = 5.735(0. 004)*	*F* = 5.143(0.007) *
Previous injury history					
No injury	31.59 ± 7.33	95.06 ± 17.83(a)	23.27 ± 11.54(a)	20.62 ± 7.77(a)	19.90 ± 7.52(a)
<3 Months	32.46 ± 8.06	87.38 ± 15.10(a)	29.43 ± 12.98(a)	26.14 ± 8.16(a)	25.22 ± 8.16(a)
3–<6 Months	30.32 ± 7.43	88.23 ± 18.35(a)	23.91 ± 10.77(a)	24.68 ± 9.06(a)	23.32 ± 8.71(a)
6–<9 Months	29.20 ± 6.14	90.00 ± 19.34(a)	23.20 ± 7.73(a)	21.60 ± 8.38(a)	19.60 ± 8.50(a)
9–<12 Months	37.71 ± 7.20	82.29 ± 10.72(a)	30.14 ± 11.87(a)	28.00 ± 6.51(a)	26.00 ± 6.63(a)
≥12 Months	33.00 ± 5.61	86.94 ± 16.01(a)	30.19 ± 11.62(a)	25.92 ± 7.76(a)	24.92 ± 7.64(a)
Test of sig. (p)	*F* = 1.514(0.187)	*F* = 2.401(0.038) *	*F* = 3.039(0.011) *	*F* = 4.695(0.000) *	*F* = 4.301(0.001) *
Sleeping hours					
Less than 4 h	27.00 ± 5.66	87.50 ± 2.12	37.50 ± 2.12	30.00 ± 2.83	29.00 ± 2.83
4–8 h	31.96 ± 7.53	90.83 ± 16.60	26.51 ± 12.33	23.16 ± 8.70	22.43 ± 8.42
More than 8 h	32.32 ± 6.30	91.82 ± 19.68	23.32 ± 10.54	23.08 ± 7.07	21.48 ± 7.02
Test of sig. (p)	*F* = 0.527(0.591)	*F* = 0.104(0.901)	*F* = 2.329(0.100)	*F* = 0.675(0.510)	*F* = 0.961(0.384)
Smoking					
Yes	34.88 ± 6.81	82.13 ± 15.88	35.00 ± 11.09	29.94 ± 6.74	27.63 ± 6.59
No	31.76 ± 7.23	91.77 ± 17.21	25.09 ± 11.76	22.65 ± 8.19	21.82 ± 8.05
Test of sig. (p)	T = 1.662(0.098)	T = 2.166(0.031) *	T = 3.252(0.001) *	T = 3.462(0.001) *	T = 2.806(0.006) *

*T*: independent samples test; *F*: one way ANOVA; *Significant *p* at ≤0.05; **Significant *p* at *p* < 0.01. Different letters (a, b) indicate statistically significant differences between groups at *p* < 0.05, whereas means sharing a common letter (e.g., ab) are not significantly different from groups labeled with either letter.

Table 5 describes the relationship between FMS and personal and academic data that demonstrates significant associations between functional movement outcomes and participants’ demographic and academic characteristics. Gender was the most consistent predictor, with females achieving higher scores across nearly all FMS components, including the Deep Squat, Hurdle Step, In-line Lunge, and Trunk Stability (all *p* < 0.01), resulting in a significantly higher total FMS score compared to males (17.22 vs. 14.52; *p* < 0.001). GPA was linked to specific components, with higher-achieving students performing better in the Deep Squat and In-line Lunge; however, overall FMS scores did not differ significantly by GPA level (*p* = 0.406), suggesting that academic performance may influence isolated abilities rather than overall movement quality. Physical activity level showed a clear positive effect, as moderately and highly active students outperformed sedentary peers in multiple components and in total FMS score (*p* < 0.001). Injury history also played an important role. Students with recent injuries (within the past 6–9 months) recorded the lowest total FMS scores, while those without injuries or with injuries over a year old performed significantly better, particularly in the Deep Squat (*p* < 0.001), In-line Lunge (*p* = 0.002), and total score (*p* = 0.048). This indicates that musculoskeletal injuries have a sustained negative impact on mobility, balance, and coordination. By contrast, age, sleep duration, and smoking status did not show significant associations with FMS performance (all *p* > 0.05).

**Table 5:** Relationship between functional movement (FMS) variables and personal and academic data.

Personal and academic data	Deep squat	Hurdle Step	In-line Lunge	Straight Leg	Trunk stability test on wooden planks	Rotational stability	Shoulder stability test	Total of FMS
	Mean ± SD.	Mean ± SD.	Mean ± SD.	Mean ± SD.	Mean ± SD.	Mean ± SD.	Mean ± SD.	Mean ± SD.
Age								
≤18 years	2.14 ± 0.72	2.51 ± 0.78	2.41 ± 0.71	2.37 ± 0.68	2.51 ± 0.80	2.56 ± 0.69	1.89 ± 0.74	16.38 ± 4.08
>18 years	1.99 ± 0.81	2.38 ± 0.76	2.34 ± 0.71	2.30 ± 0.76	2.42 ± 0.83	2.52 ± 0.69	1.97 ± 0.77	15.92 ± 4.00
Test of sig. (p)	T = 1.269(0.206)	T = 1.134(0.258)	T = 0.722(0.471)	T = 0.576(0.565)	T = 0.729(0.467)	T = 0.338(0.736)	T = 0.729(0.467)	T = 0.764(0.446)
Gender								
Male	1.71 ± 0.88	2.22 ± 0.76	2.01 ± 0.73	2.23 ± 0.72	2.19 ± 0.90	2.43 ± 0.74	1.72 ± 0.67	14.52 ± 3.79
Female	2.29 ± 0.60	2.56 ± 0.74	2.62 ± 0.57	2.39 ± 0.74	2.64 ± 0.70	2.61 ± 0.64	2.12 ± 0.78	17.22 ± 3.80
Test of sig. (p)	T = 5.623(0.000) *	T = 3.247(0.001) *	T = 6.824(0.000)*	T = 1.502(0.135)	T = 4.075(0.000) *	T = 1.800(0.073)	T = 3.841(0.000) *	T = 5.080(0.000) *
GPA								
D (<2)	1.00 ±	2.00 ±	2.00 ±	3.00 ±	3.00 ±	3.00 ±	2.00 ±	16.00 ±
C (2–<3)	1.50 ± 1.09(a)	2.42 ± 0.67	2.17 ± 0.58(a)	2.67 ± 0.49	2.58 ± 0.79	2.67 ± 0.49	1.75 ± 0.75	15.75 ± 3.39
B (3–<4)	2.02 ± 0.75(b)	2.36 ± 0.78	2.20 ± 0.74(a)	2.17 ± 0.75	2.31 ± 0.87	2.50 ± 0.69	1.97 ± 0.78	15.53 ± 4.12
A (4–5)	2.11 ± 0.75(b)	2.45 ± 0.77	2.45 ± 0.69(a)	2.36 ± 0.73	2.49 ± 0.80	2.53 ± 0.70	1.95 ± 0.76	16.34 ± 4.04
Test of sig. (p)	*F* = 3.440(0.034) *	*F* = 0.277(0.758)	*F* = 3.259(0.040)*	*F* = 2.862(0.059)	*F* = 1.228(0.295)	*F* = 0.296(0.744)	*F* = 0.430(0.651)	*F* = 0.906(0.406)
Physical activity level								
Sedentary	2.00 ± 1.00(a)	2.33 ± 0.58(a)	2.33 ± 0.58 (a)	3.00 ± 0.00(b)	2.67 ± 0.58(a)	3.00 ± 0.00(a)	2.67 ± 0.58(b)	18.00 ± 2.65(a)
Moderately active	2.13 ± 0.75(a)	2.53 ± 0.72(a)	2.46 ± 0.69(a)	2.40 ± 0.70(ab)	2.53 ± 0.77(a)	2.61 ± 0.64(a)	2.03 ± 0.78(ab)	16.68 ± 3.94(a)
Highly active	1.79 ± 0.82(a)	2.12 ± 0.83(a)	2.11 ± 0.70(a)	2.09 ± 0.79(a)	2.21 ± 0.92(a)	2.30 ± 0.76(a)	1.70 ± 0.65(a)	14.32 ± 3.80(a)
Test of sig. (p)	*F* = 4.114(0.018) *	*F* = 6.099(0.003) *	*F* = 5.290(0.006) *	*F* = 5.162(0.006) *	*F* = 3.322(0.038) *	*F* = 5.180(0.006) *	*F* = 5.337(0.005) *	*F* = 8.020(0.000) *
Previous injury history								
No injury	2.19 ± 0.71(abc)	2.53 ± 0.74	2.55 ± 0.62(a)	2.31 ± 0.70	2.54 ± 0.77	2.61 ± 0.62	1.99 ± 0.76	16.72 ± 3.79(a)
<3 Months	1.86 ± 0.82(abc)	2.24 ± 0.83	2.16 ± 0.73(a)	2.30 ± 0.74	2.41 ± 0.83	2.46 ± 0.77	1.86 ± 0.71	15.30 ± 4.29(a)
3–<6 Months	2.27 ± 0.70(bc)	2.36 ± 0.79	2.36 ± 0.66(a)	2.45 ± 0.74	2.55 ± 0.80	2.59 ± 0.67	2.14 ± 0.77	16.73 ± 4.08(a)
6–<9 Months	1.40 ± 1.14(a)	2.00 ± 1.00	2.00 ± 0.71(a)	2.00 ± 1.00	2.00 ± 1.00	1.80 ± 0.84	1.40 ± 0.89	12.60 ± 5.13(a)
9–<12 Months	2.57 ± 0.53(c)	2.29 ± 0.95	2.43 ± 0.53(a)	2.43 ± 0.79	2.14 ± 0.90	2.57 ± 0.79	2.00 ± 0.58	16.43 ± 2.88(a)
≥12 Months	1.64 ± 0.76(ab)	2.39 ± 0.69	2.06 ± 0.83(a)	2.31 ± 0.79	2.28 ± 0.91	2.44 ± 0.73	1.86 ± 0.80	14.97 ± 4.00(a)
Test of sig. (p)	*F* = 5.130(0.000)*	*F* = 1.187(0.317)	*F* = 3.894(0.002)*	*F* = 0.376(0.865)	*F* = 1.142(0.339)	*F* = 1.648(0.149)	*F* = 1.041(0.395)	*F* = 2.278(0.048)*
Sleeping hours								
Less than 4 h	2.00 ± 1.41	2.50 ± 0.71	2.50 ± 0.71	3.00 ± 0.00	3.00 ± 0.00	3.00 ± 0.00	2.50 ± 0.71	18.50 ± 3.54
4-8 h	2.02 ± 0.78	2.40 ± 0.76	2.29 ± 0.73	2.32 ± 0.71	2.43 ± 0.81	2.54 ± 0.68	1.95 ± 0.76	15.96 ± 3.98
More than 8 h	2.10 ± 0.79	2.46 ± 0.81	2.56 ± 0.61	2.28 ± 0.81	2.46 ± 0.86	2.50 ± 0.71	1.92 ± 0.78	16.28 ± 4.19
Test of sig. (p)	*F* = 0.203 (0.816)	*F* = 0.122(0.885)	*F* = 2.789(0.064)	*F* = 0.940(0.392)	*F* = 0.481(0.619)	*F* = 0.518(0.597)	*F* = 0.558(0.573)	*F* = 0.494(0.611)
Smoking								
Yes	1.88 ± 0.89	2.31 ± 0.70	2.25 ± 0.68	2.44 ± 0.73	2.38 ± 0.81	2.63 ± 0.62	1.88 ± 0.72	15.75 ± 3.55
No	2.05 ± 0.78	2.42 ± 0.77	2.37 ± 0.71	2.31 ± 0.73	2.45 ± 0.82	2.52 ± 0.69	1.95 ± 0.77	16.08 ± 4.06
Test of sig. (p)	T = 0.867(0.387)	T = 0.561(0.575)	T = 0.640(0.523)	T = 0.664(0.508)	T = 0.355(0.723)	T = 0.568(0.570)	T = 0.395(0.693)	T = 0.318(0.751)

FMS: functional movement screen; T: independent samples test; F: one way ANOVA; *Significant *p* at ≤0.05; **Significant *p* at *p* < 0.01. Different letters (a, b) indicate statistically significant differences between groups at *p* < 0.05, whereas means sharing a common letter (e.g., ab) are not significantly different from groups labeled with either letter.

The regression analysis Table 6 describe general linear model (GLM) Parameter which highlights gender as the strongest predictor of FMS scores, with males showing significantly lower outcomes compared to females (*B* = −2.557; *p* < 0.001). Physical activity also played a key role, where moderate activity was associated with higher FMS scores than high activity (*B* = 2.014; *p* = 0.002), suggesting that balanced exercise may better support movement quality than excessive training. Injury history and sleep duration showed limited but notable effects. Participants injured 3–6 months prior had higher FMS scores (*B* = 2.064; *p* = 0.041), likely reflecting improved post-rehabilitation mechanics. Also, very short sleep (<4 h) appeared as a negative predictor (*B* = −6.334; *p* = 0.037). Other factors, including age, GPA, smoking, and older injuries, were not significant predictors of functional movement quality. The goodness-of-fit statistics indicated that the GLM provided an acceptable representation of the data. The deviance value was 2,550.783 (df = 185; Deviance/df = 13.788), and the Pearson chi-square statistic showed a similar ratio (*χ*^2^ = 2,550.783; *χ*^2^/df = 13.788), suggesting the presence of dispersion in the data. After applying the scale adjustment, the scaled deviance and scaled Pearson chi-square values were 209.000 with 185 degrees of freedom, indicating that the dispersion was adequately accounted for and that the model demonstrated a satisfactory fit. The model produced a log-likelihood of −557.998, with information criteria values of AIC = 1,165.997, AICC = 1,173.101, BIC = 1,249.555, and CAIC = 1,274.555, supporting the adequacy of the model in explaining variability in total FMS based on the included predictors.

**Table 6:** General linear model (GLM) parameter estimates and significance tests for predictors of functional movement screen (FMS) score based on personal and academic variables.

Parameter estimates							
Parameter	*B*	Std. Error	95% Wald confidence interval		Hypothesis test		
			Lower	Upper	Wald chi-square	df	Sig.
(Intercept)	16.254	3.0728	10.232	22.277	27.982	1	0.000
[Age = 1.00]	−0.367	0.5652	−1.475	0.741	0.422	1	0.516
[Age = 2.00]	0^a^						
[Gender = 1.00]	−2.557	0.6809	−3.891	−1.222	14.102	1	0.000
[Gender = 2.00]	0^a^						
[Academic Grads = 1.00]	0.272	0.9981	−1.685	2.228	0.074	1	0.785
[Academic Grads = 2.00]	3.690	2.2208	−0.663	8.043	2.761	1	0.097
[Academic Grads = 3.00]	−4.496	2.5130	−9.422	0.429	3.201	1	0.074
[Academic Grads = 4.00]	0^a^						
[GPA = 1.00]	−3.462	4.2276	−11.748	4.824	0.671	1	0.413
[GPA = 2.00]	0.835	1.2335	−1.583	3.253	0.458	1	0.499
[GPA = 3.00]	−0.498	0.5692	−1.614	0.617	0.766	1	0.382
[GPA = 4.00]	0^a^						
[Marital status = 1.00]	−1.220	2.6416	−6.398	3.957	0.213	1	0.644
[Marital status = 2.00]	−2.151	3.0471	−8.123	3.821	0.498	1	0.480
[Marital status = 3.00]	0^a^						
[Physical Activity Level = 1.00]	2.810	2.5108	−2.111	7.731	1.253	1	0.263
[Physical Activity Level = 2.00]	2.014	0.6523	0.736	3.293	9.537	1	0.002
[Physical Activity Level = 3.00]	0^a^						
[Sports Participation = 1.00]	0.789	0.6614	−0.508	2.085	1.422	1	0.233
[Sports Participation = 2.00]	0^a^						
[Previous Injury History = 1.00]	1.163	0.7340	−0.276	2.601	2.508	1	0.113
[Previous Injury History = 2.00]	1.013	0.9150	−0.781	2.806	1.225	1	0.268
[Previous Injury History = 3.00]	2.064	1.0082	0.088	4.040	4.191	1	0.041
[Previous Injury History = 4.00]	−2.698	1.7172	−6.064	0.667	2.469	1	0.116
[Previous Injury History = 5.00]	1.500	1.5345	−1.507	4.508	0.956	1	0.328
[Previous Injury History = 6.00]	0^a^						
[Sleeping hours = 1.00]	- 6.334	3.0390	0.378	12.291	4.345	1	0.037
[Sleeping hours = 2.00]	−0.154	0.5880	−1.306	0.999	0.068	1	0.794
[Sleeping hours = 3.00]	0^a^						
[Eating patterns = 1.00]	−0.015	0.5381	−1.070	1.039	0.001	1	0.977
[Eating patterns = 2.00]	0^a^						
[Smoking = 1.00]	0.905	1.0899	−1.231	3.041	0.689	1	0.406
[Smoking = 2.00]	0^a^						
[Do you use the internet for work purposes? = 1.00]	−1.047	0.8969	−2.805	0.711	1.362	1	0.243
[Do you use the internet for work purposes? = 2.00]	0^a^						
(Scale)	12.205^b^	1.1939	10.075	14.784			

*The mean of the standardized scores was calculated (T score) for FMS to perform the statistical analysis; *Significant *p* ≤ 0.05; **Significant *p* at *p* < 0.01.

The regression analysis of physical fitness (T-scores) in Table 7 describes general linear model (GLM) parameter estimates and significance tests for predictors of physical fitness score based on personal and academic variables that confirmed the model’s validity and identified gender, age, and GPA as the strongest predictors. Gender had the largest effect, with males demonstrating significantly higher fitness scores than females (B = 13.662; *p* < 0.001), consistent with earlier descriptive findings. Age also contributed positively, as participants under 18 exhibited lower fitness levels (*B* = 1.926; *p* = 0.006), reflecting the influence of physical maturation on strength and endurance. Academically, a very low GPA (<2.0) was strongly associated with reduced fitness (*B* = −14.998; *p* = 0.004), suggesting that poor academic achievement may parallel lower physical health. In contrast, physical activity level did not significantly predict physical fitness (*p* > 0.05), highlighting a distinction from its effect on FMS, where movement quality was more activity-dependent. Similarly, injury history, sleep duration, and smoking status were not significant predictors, indicating that intrinsic factors such as sex, age, and academic performance exert stronger independent effects on overall physical fitness. The goodness-of-fit statistics indicated that the GLM provided an acceptable representation of the observed data for Physical Fitness. The deviance value was 3,905.426 (df = 185; Deviance/df = 21.110), and the Pearson chi-square statistic showed a similar pattern (*χ*^2^ = 3905.426; *χ*^2^/df = 21.110), suggesting the presence of dispersion in the data. After applying the scale correction, both the scaled deviance and scaled Pearson chi-square values were 209.000 with 185 degrees of freedom, indicating that the dispersion was adequately adjusted and that the model achieved a satisfactory level of agreement with the observed data. The model produced a log-likelihood value of −602.512, while the information criteria were AIC = 1,255.024, AICC = 1,262.128, BIC = 1,338.582, and CAIC = 1,363.582, supporting the adequacy of the model and indicating that the included demographic and lifestyle predictors collectively provide substantial explanatory capability for variations in Physical Fitness.

**Table 7:** General linear model (GLM) parameter estimates and significance tests for predictors of physical fitness score based on personal and academic variables.

Parameter estimates							
Parameter	*B*	Std. error	95% Wald confidence interval		Hypothesis test		
			Lower	Upper	Wald chi-square	df	Sig.
(Intercept)	45.042	3.8022	37.590	52.494	140.339	1	0.000
[Age = 1.00]	1.926	0.6993	0.555	3.296	7.583	1	0.006
[Age = 2.00]	0^a^						
[Gender = 1.00]	13.662	0.8425	12.010	15.313	262.930	1	0.000
[Gender = 2.00]	0^a^						
[Academic Grads = 1.00]	−0.004	1.2350	−2.425	2.416	0.000	1	0.997
[Academic Grads = 2.00]	2.020	2.7480	−3.366	7.406	0.540	1	0.462
[Academic Grads = 3.00]	−0.960	3.1094	−7.055	5.134	0.095	1	0.757
[Academic Grads = 4.00]	0^a^						
[GPA = 1.00]	−14.998	5.2311	−25.251	−4.745	8.220	1	0.004
[GPA = 2.00]	0.844	1.5263	−2.148	3.835	0.306	1	0.580
[GPA = 3.00]	0.298	0.7043	−1.083	1.678	0.179	1	0.673
[GPA = 4.00]	0^a^						
[Marital status = 1.00]	−2.173	3.2686	−8.579	4.233	0.442	1	0.506
[Marital status = 2.00]	−3.141	3.7703	−10.531	4.249	0.694	1	0.405
[Marital status = 3.00]	0^a^						
[Physical Activity Level = 1.00]	3.159	3.1068	−2.931	9.248	1.034	1	0.309
[Physical Activity Level = 2.00]	0.609	0.8071	−0.973	2.191	0.569	1	0.451
[Physical Activity Level = 3.00]	0^a^						
[Sports Participation = 1.00]	0.439	0.8184	−1.165	2.043	0.288	1	0.592
[Sports Participation = 2.00]	0^a^						
[Previous Injury History = 1.00]	−0.614	0.9083	−2.394	1.166	0.457	1	0.499
[Previous Injury History = 2.00]	−1.442	1.1321	−3.661	0.777	1.623	1	0.203
[Previous Injury History = 3.00]	−0.675	1.2475	−3.120	1.770	0.293	1	0.589
[Previous Injury History = 4.00]	−3.465	2.1248	−7.630	0.699	2.660	1	0.103
[Previous Injury History = 5.00]	−2.790	1.8987	−6.511	0.932	2.159	1	0.142
[Previous Injury History = 6.00]	0^a^						
[Sleeping hours = 1.00]	−0.231	3.7603	−7.601	7.139	0.004	1	0.951
[Sleeping hours = 2.00]	0.991	0.7276	−0.436	2.417	1.853	1	0.173
[Sleeping hours = 3.00]	0^a^						
[Eating patterns = 1.00]	−0.611	0.6658	−1.916	0.694	0.842	1	0.359
[Eating patterns = 2.00]	0^a^						
[Smoking = 1.00]	0.370	1.3486	−2.273	3.013	0.075	1	0.784
[Smoking = 2.00]	0^a^						
[Do you use the internet for work purposes? = 1.00]	0.163	1.1098	−2.012	2.338	0.021	1	0.883
[Do you use the internet for work purposes? = 2.00]	0^a^						
(Scale)	18.686^b^	1.8279	15.426	22.635			

*The mean of the standardized scores was calculated (T score) for Physical Fitness to perform the statistical analysis;* Significant *p* ≤ 0.05, **Significant *p* at *p* < 0.01.

## Discussion

This study investigated the predictive roles of gender, physical activity level, and sleep duration on FMS scores and physical fitness outcomes among university students. The first major finding revealed that gender significantly predicted both functional movement and physical fitness outcomes, thereby supporting H1. Specifically, female students outperformed males in functional movement tasks, reflecting superior mobility, balance, and motor control, whereas males demonstrated higher overall physical fitness, particularly in strength and endurance. These outcomes highlight the distinct physiological and biomechanical differences between genders, which influence movement efficiency and physical capacity.

The findings of this study provide a multidimensional perspective on functional movement and physical fitness among non-athletic university students. By simultaneously examining gender, physical activity level, and sleep duration, we extend existing knowledge beyond single-factor investigations that have largely focused on athletic populations. Consistent with prior research, gender differences in strength and flexibility were observed, and higher physical activity levels were associated with better functional movement. However, the integration of multiple predictors highlights how these factors interact within a university population, offering a more comprehensive understanding of the determinants of physical fitness.

This approach has practical implications for health promotion and intervention design. For instance, fitness programs can be tailored to account for gender-specific movement patterns, varying activity levels, and the influence of sleep on performance and recovery. Additionally, the identification of at-risk subgroups supports targeted strategies to improve functional movement and reduce injury risk. By framing these findings within a holistic, biopsychosocial context, this study contributes meaningful insights to the literature on student health and informs interventions aimed at enhancing both physical performance and overall well-being in academic settings.

Prior studies support these findings. Pérez et al. (23) found that university-aged men demonstrated significantly greater handgrip strength and countermovement jump performance than women, a disparity largely attributable to differences in lean body mass. Similar findings have been reported in broader physiological assessments, with men consistently displaying substantially higher grip strength and muscular power (24). In contrast, several studies suggest that females may excel in movement quality tasks: research on functional movement patterns in adolescents revealed that girls scored higher than boys on FMS composite scores, especially in flexibility-related components such as the straight-leg raise (25). Notably, some studies offer nuanced or even opposing perspectives: Thomas et al. (26) observed no significant difference in total FMS scores between male and female collegiate tennis players, though females performed better in flexibility (inline lunge, active straight-leg raise) and males in upper-body strength tasks. Together, these findings indicate that while gender is a robust predictor of both FMS and physical fitness outcomes, its influence can vary depending on specific movement tasks, training history, and athletic background.

The second key finding demonstrated that higher levels of physical activity were positively associated with improved functional movement and physical fitness performance compared to sedentary or moderately active students, thereby supporting H2. This highlights the critical role of regular physical activity in enhancing neuromuscular coordination, joint stability, cardiovascular efficiency, and overall movement quality. Consistent with the present results, Bonazza et al. (27) found that physically active individuals achieved significantly higher FMS scores, suggesting that habitual exercise promotes better mobility and movement efficiency. Similarly, Muehlbauer et al. (24) reported that youth athletes with higher training volumes consistently demonstrated superior FMS scores compared to their less active peers, reinforcing the link between physical activity and movement quality. Beyond movement efficiency, research has shown that higher physical activity levels contribute to superior strength, aerobic fitness, and body composition, which directly translate into improved performance outcomes (28). However, not all studies fully align with these findings. For example, Rangel Caballero et al. (29) observed that among collegiate athletes, differences in FMS scores were not solely explained by physical activity levels but were also influenced by sport-specific training demands and motor learning experiences. Likewise, Teyhen et al. (30) suggested that while physical activity improves overall conditioning, FMS outcomes are multifactorial and may depend on technique, mobility deficits, or injury history. Taken together, these findings underscore that while higher physical activity is a robust predictor of better functional movement and physical fitness, its influence may be moderated by factors such as sport specialization, training quality, and musculoskeletal health.

In our sample, sleep duration did not significantly predict FMS performance, so H3 was not supported. This null finding may reflect the cohort’s narrow age range, limited between-person variability in habitual sleep, reliance on self-reported sleep, and overshadowing effects from stronger predictors (e.g., gender and activity level). The broader literature generally links insufficient or poor-quality sleep to worse athletic outcomes, lower physical performance and recovery, and higher injury risk mechanisms that plausibly influence movement quality (e.g., impaired neuromotor control, attentional lapses, and reduced tissue recovery) (31). Narrative and systematic reviews show that suboptimal sleep is associated with decrements in performance and an elevated injury risk in youth and adult athletes, and highlight ≥7–8 h/night as a practical benchmark for student-athletes (11, 31).

At the sensorimotor level, experimental sleep loss impairs postural control, especially under challenging sensory conditions, supporting a pathway by which inadequate sleep could degrade functional movement; still, effects can be task- and context-dependent (32). By contrast, some controlled work in physically active adults observed minimal deterioration in postural stability after acute sleep deprivation, suggesting that trained individuals may partially compensate, which aligns with our null association for FMS (33). Overall, existing evidence implies that sleep can affect factors related to FMS (injury susceptibility, balance under constraint), yet direct associations with composite FMS scores remain inconsistent; future studies using objective sleep measures, larger extremes of sleep duration, and task-specific balance/mobility probes may clarify these relationships.

The fourth key finding demonstrated that previous injury history negatively influenced functional movement performance, with students who experienced a recent injury (<6 months) scoring significantly lower on FMS compared to their uninjured peers. This outcome supports H4 and underscores the critical role of prior musculoskeletal injuries in compromising movement quality, neuromuscular control, and overall functional performance. Injuries often lead to residual deficits such as joint instability, altered biomechanics, and compensatory movement patterns, which can persist long after initial recovery and contribute to reduced FMS scores (34). These findings align with a growing body of evidence suggesting that FMS is a sensitive tool for detecting lingering functional deficits post-injury. For example, Mokha et al. (35) reported that collegiate athletes with a history of lower-extremity injuries exhibited poorer FMS composite scores compared to uninjured counterparts. Similarly, Vernadakis et al. (36) found that athletes with previous injuries demonstrated higher asymmetry rates in FMS tasks, highlighting the long-term impact of musculoskeletal trauma on movement efficiency.

However, not all studies report uniform effects. Dorrel et al. (37) observed that when athletes undergo structured rehabilitation and neuromuscular training, the negative impact of prior injuries on FMS performance may be attenuated, suggesting that effective recovery strategies can mitigate these deficits. Furthermore, Bonazza et al. (27), in a systematic review, emphasized that while injury history is strongly associated with lower FMS scores and higher risk of re-injury, the predictive validity of FMS is influenced by contextual factors such as sport type, rehabilitation adherence, and training exposure.

Some findings, such as smokers demonstrating higher fitness levels and sedentary participants achieving higher-than-expected push-up scores, were counterintuitive. These results may be influenced by small subgroup sizes, imbalanced category distributions, or sampling variability (38, 39). Therefore, these findings are presented as exploratory and potentially unstable, and are interpreted conservatively rather than mechanistically. Readers are cautioned that these observations may not reflect generalizable patterns, and further research with larger and more balanced samples is needed to confirm these associations (40, 41).

While these findings provide interesting insights, they may not reflect true physiological or behavioural patterns and highlight the need for caution in generalization. Future studies with larger and more balanced samples, as well as objective assessments of lifestyle factors, are needed to verify these associations and clarify underlying mechanisms. Collectively, these findings reinforce the notion that injury history remains a significant determinant of functional movement performance, but also highlight the importance of comprehensive rehabilitation and targeted preventive training programs in restoring optimal neuromuscular function and reducing long-term deficits.

### Interpretation of associations

Given the cross-sectional design of this study, all observed relationships between gender, physical activity level, sleep duration, and functional movement or physical fitness outcomes should be interpreted as associative rather than causal. While the findings provide valuable insights into factors linked with functional movement and fitness in university students, the temporal order of these relationships cannot be established. Therefore, the results indicate correlation, not causation, and future longitudinal or intervention-based studies are warranted to explore causal pathways and confirm these associations.

Finally, this study confirms that gender, physical activity, sleep, and recent injury history are critical predictors of functional movement and physical fitness in university students. These results emphasize the need for targeted interventions that promote regular activity, adequate rest, and effective injury prevention to optimize student health and performance.

### Recommendations

Based on the findings, it is recommended that physical education and health programs adopt a more individualized and evidence-based approach. Gender-specific training protocols should be implemented to address the unique strengths and limitations observed, such as incorporating mobility and neuromuscular control training for males and upper-body strength conditioning for females. The FMS should be integrated as a preventive assessment tool within athletic, academic, and health monitoring frameworks, allowing for early identification of compensatory movement patterns before they lead to injury. Additionally, moderate levels of physical activity should be emphasized, as they were associated with optimal functional movement outcomes. Students with recent injury history should not be rushed back into training but instead follow a structured rehabilitation and reassessment pathway to restore full movement integrity. Institutions should also promote a holistic balance between academic demands and physical activity, especially for high-achieving students who may be at risk of muscular deconditioning. While sleep and smoking showed limited predictive value in this dataset, they should still be monitored as part of broader wellness policies due to their known long-term health implications.

### Implications for practice

The results carry several practical implications for educators, health professionals, and institutional policymakers. For academic institutions, integrating functional movement and fitness education into the curriculum can enhance students’ physical literacy and reduce injury risk. Physical educators and coaches can use these insights to tailor training programs more effectively, focusing not only on performance but also on movement efficiency and injury prevention. Rehabilitation professionals should be particularly attentive to the post-injury window of 3 to 9 months, where movement deficits remain significantly impaired, even if physical strength appears to have returned. Furthermore, policy-makers can use this data to advocate for routine FMS assessments and customized intervention strategies, particularly for at-risk subgroups. The clear association between moderate physical activity and functional competence underscores the need for sustainable, non-extreme activity promotion across university settings. Ultimately, these findings support a more integrated, data-driven approach to student health and performance, bridging physical, academic, and behavioral domains in a holistic model of human development.

### Limitations and future directions

This study has several limitations that should be considered when interpreting the findings. The cross-sectional design precludes causal inferences, while the single-university convenience sample limits generalizability to broader student populations. Small subgroup sizes (e.g., sedentary students, smokers) produced unstable estimates, and reliance on self-reported lifestyle factors (physical activity, sleep duration) may have introduced recall and social desirability bias. Additionally, unmeasured confounders such as nutritional status, psychological stress, and socioeconomic factors were not controlled for, and the lack of assessor blinding may have introduced measurement bias. To address these limitations, future research should employ longitudinal or intervention-based designs to establish causality, recruit multi-center samples across diverse institutions and cultural contexts to enhance generalizability, and use objective measures (e.g., accelerometers for physical activity, polysomnography for sleep, direct VOC max testing for cardiovascular fitness) to improve measurement accuracy. Furthermore, researchers should explore interaction effects between predictors, incorporate additional fitness components (body composition, agility, power), and validate findings using more sensitive movement assessment tools to better understand the complex relationships between gender, physical activity, sleep, injury history, and functional movement in university populations.

## Conclusion

This study highlights the complex interplay between physical fitness, functional movement, and personal characteristics among university students. Gender emerged as the strongest predictor, with females demonstrating superior movement quality and males excelling in strength and endurance. Physical activity significantly enhanced functional movement but was less influential for overall fitness, while recent injuries impaired movement efficiency. In contrast, sleep duration and smoking status were not significant predictors when stronger factors were considered. These findings emphasize the need for integrated strategies that address gender-specific strengths, promote regular physical activity, and prioritize injury prevention to support student health and performance.

## Data Availability

The original contributions presented in the study are included in the article/supplementary material, further inquiries can be directed to the corresponding author.
